# Local Delivery and Glioblastoma: Why Not Combining Sustained Release and Targeting?

**DOI:** 10.3389/fmedt.2021.791596

**Published:** 2021-11-22

**Authors:** Claire Gazaille, Marion Sicot, Patrick Saulnier, Joël Eyer, Guillaume Bastiat

**Affiliations:** Univ Angers, Inserm, CNRS, MINT, SFR ICAT, Angers, France

**Keywords:** glioblastoma, Gliadel^®^ wafers, local delivery, nanoparticle-loaded hydrogel, targeting

## Abstract

Glioblastoma is one of the most aggressive brain tumors and is associated with a very low overall median survival despite the current treatment. The standard of care used in clinic is the Stupp's protocol which consists of a maximal resection of the tumor when possible, followed by radio and chemotherapy using temozolomide. However, in most cases, glioblastoma cells infiltrate healthy tissues and lead to fatal recurrences. There are a lot of hurdles to overcome in the development of new therapeutic strategies such as tumor heterogeneity, cell infiltration, alkylating agent resistance, physiological barriers, etc., and few treatments are on the market today. One of them is particularly appealing because it is a local therapy, which does not bring additional invasiveness since tumor resection is included in the gold standard treatment. They are implants: the Gliadel^®^ wafers, which are deposited post-surgery. Nevertheless, in addition to presenting important undesirable effects, it does not bring any major benefit in the therapy despite the strategy being particularly attractive. The purpose of this review is to provide an overview of recent advances in the development of innovative therapeutic strategies for glioblastoma using an implant-type approach. The combination of this local strategy with effective targeting of the tumor microenvironment as a whole, also developed in this review, may be of interest to alleviate some of the obstacles encountered in the treatment of glioblastoma.

## Introduction

Glioblastoma (GBM) is the most common primary brain tumor in adults. It is characterized by high infiltration into healthy brain tissue, rapid proliferation and important intra- and inter-tumor heterogeneity leading to a global chemoresistance and a high aggressivity (1). This brain tumor is classified as a grade IV glioma tumor by the World Health Organization (2). Despite a still low incidence (3.23 per 100,000 population) and a glioblastoma primarily diagnosed at older ages (65 years), the median survival for glioblastoma for all patients (regardless of treatment) was 8 months in the United States, with a one-, five- and ten-year survival rates of 42.8, 7.2 and 4.7 %, respectively, based on recent statistical analysis of the Central Brain Tumor Registry of the United States (CBTRUS) (3, 4).

Approved FDA treatments against GBM are scarce primarily due to the difficulty of treating brain pathologies because of the blood-brain barrier (BBB) that protects the central nervous system (5). In systemic regimen, only a few therapeutic molecules have the capacity to cross the barrier to reach the tumor areas to be treated. Lomustine (CCNU) approved in 1976 for oral administration (80–110 mg/m^2^ every 6 weeks) and carmustin (BCNU) approved in 1977 for intravenous administration (150–200 mg/m^2^ every 6 weeks) are non-specific alkylating agents that causes crosslinking of DNA and RNA in dividing cells, triggering cell death (6, 7). There is no benefit for these treatments compared to radiotherapy alone, and high adverse effects such as hematologic toxicity for CCNU and bone marrow suppression, pulmonary and ocular toxicities for BCNU. Another systemic treatment approved in 2009 consists of a targeted therapeutic antibody: Bevacizumab (intravenous administration, 10 mg/kg every 2 weeks) (8). With strong side effects such as hypertension, thromboembolic events, gastrointestinal perforation, cerebral bleeding, proteinuria, this treatment is used to treat symptomatic edema and radiation necrosis.

The last systemic treatment approved in 2005 is temozolomide (TMZ), another non-specific alkylating agent that causes mismatch repair in DNA with the methylation of the O6 position of guanine. This treatment is part of the standard of care of GBM: the Stupp's protocol, including maximal tumor resection by neurosurgery, when accessible, followed by radiation therapy [Total of 60 Gy over 6 weeks: 2 Gy/day (5days/week)] and TMZ (oral administration, 75 mg/m^2^ per day for 6 weeks (concomitant to radiotherapy) followed by 120 mg/m^2^ the first 5 days of each 4 weeks, with a total of 6 cycles). This treatment presents also systemic toxicities: hematologic toxicity, thrombocytopenia, leukopenia, neutropenia, but more moderated than the previous ones (9). Among all concomitant and/or adjuvant approved therapeutic agents, TMZ led to the best therapeutic efficacy in clinic with a median survival of 14.6 months with a 5 year survival rate of 10%, confirmed by a recent meta-analysis (10). Nevertheless, this standard of care does not allow elimination of GBM since tumor recurrence occurs in most cases and in the vicinity of the tumor resection area (11). GBM is one of the most expensive cancers to treat (12), which is even more irrational since this tumor remains incurable to this day.

Because crossing the BBB is still a real challenge despite all the efforts made such as the use of drug delivery nanosystems, scientists have also explored other strategies to improve therapeutic efficacy and three of them have been approved by the FDA. The intraoperative imaging agent: 5-aminolevulinic acid (5-ALA) recently approved in 2017, allows the better visualization of GBM and other malignant glioma tissue during surgery (13). A clinical trial in 2006 showed more complete resections using fluorescence-guided surgery with 5-ALA in comparison to conventional white light microsurgery (14). Better surgical procedures combined with Stupp protocol can only be beneficial to patients in order to limit tumor recurrence. Also, in complement to the Stupp protocol, tumor treatment fields were approved by the FDA in 2011 (15). It consists of the application of an alternating electric field (low intensity: 1–3 V/cm and low frequency: 200 kHz) using electrodes on patient scalps to disrupt mitosis in tumor cells. Despite promising results in terms of overall survival (16), this therapeutic strategy was not included in the standard of care due to marginal benefits, expensive costs and inconvenience for patients (17).

The final FDA approved therapeutic approach is the Gliadel^®^ wafers. The development of post-operative implants is particularly appealing in comparison to standard chemotherapies. The use of implant in this case is minimally invasive since surgery, when possible, is part of the standard of care of patients. They make it possible to avoid the BBB crossing (so all the chemotherapeutic agents could be considered) and to have a reservoir of active molecules close to the pathology. All the active molecules will have limited systemic toxicity since they are locally administered. Nevertheless, as developed below in this review, the Gliadel^®^ wafers have limitations that make their use more and more limited, especially in Europe. However, these limitations mainly due to the stiffness of the wafers and the lack of specificity of the active molecule of Gliadel^®^ wafers: BCNU, could be overcome combining new types of implants for local delivery and active targeting. The purpose of this review is to report the recent preclinical advances that have been made on post-resection GBM implants and the targeting strategies that could be used in combination with these therapeutic implants.

## The Case of Gliadel^®^ Wafers

Gliadel^®^ wafers are currently the only implantable medicine to be granted with a marketing authorization (1998) and to be indicated in the treatment of newly diagnosed or recurrent glioblastoma. The implant consists of a slow degrading 1,3-bis-(p-carboxyphenoxy)-propane copolymer and fast degrading sebacic acid with a ratio of 20/80 (*w/w*) (Polifeprosan 20). This copolymer associated with BCNU is developed as microspheres, compressed into 1.4 cm wide and 1 mm thick disks (18, 19). The integration of Carmustin in an implant directly installed in the tumor cavity avoids the crossing of the BBB and allows for higher drug concentrations close to tumor cells.

A first phase II study on 21 patients with recurrent glioblastomas has been conducted between 1987 and 1988 (18). This study demonstrated that the effective dose of BCNU to administrate to observe survival improvement is 7.7 mg of drug per implant and 8 implants maximum (representing a maximal dose of 61.6 mg locally administrated). A phase III study has next been conducted between 1989 and 1992 (20). In total, 145 patients with recurrent glioblastoma have been recruited for this study: 73 patients were treated with placebo implants and 72 with BCNU implants. Six months post-implantation, 56% of patients treated with BCNU implants were alive compared to 36% of placebo-treated patients. Other studies were conducted on patients with newly diagnosed glioblastoma (21, 22). Between 1997 and 1999, 101 patients were treated with BCNU implants and 106 with placebo implants. Median survival was of 11.4 months for placebo-treated patients and 13.5 months for BCNU-treated patients (22). Different treatment combinations were then tested on newly diagnosed patients. A 1997 to 2006 study showed that the median survival of patients treated with Gliadel^®^ combined with Stupp's protocol (33 patients) is higher (20.7 months) than the median survival of patients only treated with Stupp's protocol (14.7 months) (45 patients). In addition, patients treated with Gliadel^®^ combined with Stupp's protocol and whose age was inferior or equal to 70 years old (30 patients) have a median survival of 21.3 months compared to 12.4 months for patients treated with Gliadel^®^ associated with radiotherapy (112 patients) (23).

However, after implantation several side effects have been observed: seizures, intracranial hypertension, meningitis, cerebral edemas, and poor wound healing (24, 25) (Food and Drug Administration. Reference ID 3358686. Available at: https://www.fda.gov/). Some of these complications could be linked to the implant characteristics. Indeed, the rigid Gliadel^®^ implant could induce micro-tears when moving after the implantation (26). These micro-tears could alter junctions between cells of the BBB leading to vasogenic edemas (27, 28). Furthermore, the use of Gliadel^®^ implants must be limited to areas not in direct contact with the ventricular system because they could migrate and induce an obstructive hydrocephalus. Finally, the implant content could release itself in a non-continuous fashion depending on the intracranial conditions, leaving degradation products behind. BCNU is supposed to be released during approximately 3 weeks; however *in vivo* studies have shown that 74% of the drug is released in the first 7 days post-implantation (29). In addition, using preclinical models, BCNU diffusion from the implant is limited to 3–12 mm in the surrounding tissues (24, 30, 31). *In vitro*, polifeprosan 20 showed an approximate 60% degradation after 6 weeks but few studies were carried on the total degradation of the implants *in vivo* (19, 29). In patients, more than 70% of copolymer is degraded in the 3 weeks following the implantation. However, reoperations and autopsies showed that polymer can stay up to 232 days after implantation (Food and Drug Administration. Reference ID 3358686. Available at: https://www.fda.gov/).

Even though Gliadel^®^ is a safer and a more effective strategy than intravenous administration of BCNU (19, 32), and numerous clinical trials proved the efficacy of the combination of Gliadel^®^ and Stupp's protocol against newly diagnosed GBM (33), its clinical use remains controversial due to the risk/benefit/cost balance (25, 27). This system is less and less used in Europe and is not ideal for GBM therapy. Finally, a recently published clinical trial has shown that surgical improvements in GBM resection limit or even negate the benefits of Gliadel^®^ wafers, without reducing its adverse effects. Median survival of patients with or without Gliadel^®^ wafers following fluorescence (5-ALA)-guided surgery did not differ significantly (14.2 and 14.3 months, respectively), and those who received the Gliadel^®^ wafers tended to have more wound infection (incidence of 15.4 vs. 7.1%) (34).

**Figure 1:**
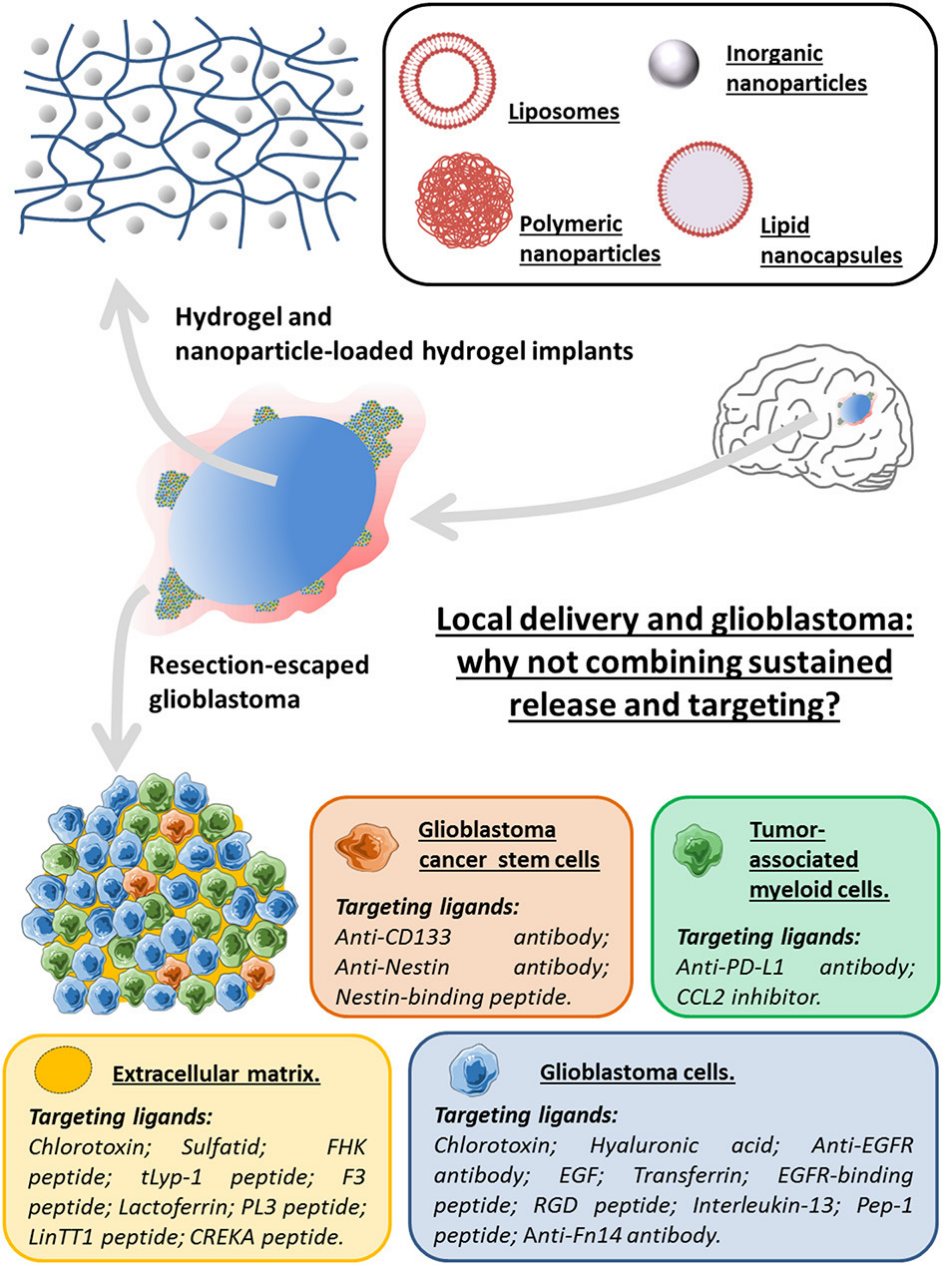
Local delivery and glioblastoma: why not combining sustained release and targeting for the design of innovative therapeutic strategies using an implant-type approach. The synergy of the local strategy using hydrogels or nanoparticles (liposomes, lipid nanocapsules or inorganic and polymeric nanoparticles)-loaded hydrogels, with ligands specific to targets being overexpressed in various elements of the GBM microenvironment as a whole (glioblastoma cells, glioblastoma cancer stem cells, tumor-associated myeloid cells and extracellular matrix) may be of interest to alleviate some of the obstacles encountered in the treatment of glioblastoma.

Establishing a local treatment directly on the tumor site remains a very promising solution to improve drug delivery. A meta-analysis directed by Bastiancich et al. (35) proved that the greater efficacy of the local administration than the systemic one, regardless of the drugs, drug delivery systems and *in vivo* preclinical models. Although not sufficient to eliminate all cancer cells, resection of the tumor creates a cavity into which the neurosurgeon can directly introduce an implant containing a therapeutic drug. Unlike a liquid solution injected into the cavity, the implant will allow a sustained release of the drug to the remaining tumor cells. So the Gliadel^®^ wafers being the only FDA approved implants can serve as a case study, and its drawbacks must be overcome in order to improve local and safe delivery strategies. Among these weaknesses, the stiffness of the implant can be reduced by using a hydrogel whose viscoelastic properties can be adapted to the host tissue: the brain. Moreover, the lack of specificity of the active molecules can be reduced by using drug delivery systems able to specifically target the GBM cells and their microenvironment as a whole (Figure 1).

## Hydrogels and Nanoparticle-Loaded Hydrogels

Hydrogels are three-dimensional structures formed by covalent or non-covalent reticulations, i.e., chemical or physical ones, respectively, of hydrophilic synthetic or natural polymers in aqueous environment (36). The diversity of the nature of polymers gives the hydrogels different properties such as bio-adhesiveness, biocompatibility, biodegradability which makes them excellent subjects for long-term use on biological tissues (37). In addition, the nature and distribution of the reticulations in the hydrogels offer a wide variation in viscoelastic and stiffness properties, some of which are compatible with administration in the brain (38), as well as greater or lesser stabilities, allowing modulation of drug release over time (39).

Hydrogels can be used in different applications like tissue engineering and wound reparation, cell culture and drug delivery (37, 39, 40). Molecules like proteins, DNA, or hydrophilic or slightly lipophilic active pharmaceutical compounds can be inserted in the hydrogel matrix (41–43). For example, Adhikari et al. (44) entrapped TMZ in an amphiphilic polylysine and polyleucine (K180L20) diblock copolypeptide hydrogel. The system gels at body temperature after the administration of the solution of copolypeptide. No toxicity of hydrogel without TMZ was observed *in vitro* on human astrocytes (NHA cell line) and neurospheres of patient GBM cells (GBM001). *In vivo*, the injection of hydrogels with or without TMZ did not induce cellular death or important inflammatory responses. On a human GBM orthotopic xenograft model (GBM001), an important decrease of the tumor volume was observed when the tumors were treated with the hydrogel combined with TMZ compared to the hydrogel without TMZ and to TMZ without hydrogel. This decrease was correlated to the increase of the median survival: 38, 20 and 28 days, respectively. The increase of TMZ effectiveness is supposed to be due to its sustained release, not evaluated in this study. Schiaparelli et al. associated camptothecin with an amphiphilic peptide (45). The amphiphilic peptide can auto-associate in approximately 10 nm wide and few μm-long filaments thanks to hydrophobic interactions and intramolecular hydrogen bonds, and this fibrillary network formed the scaffold of the hydrogel. After 30 days, only 17% of camptothecin were released *in vitro* and no burst effect was observed. This hydrogel showed its effectiveness on an orthotopic human GBM xenograft model (GBM1A-GFP-LUC) by delaying the apparition of tumor recurrence and increasing the median survival (64 days), compared to the hydrogel without camptothecin (median survival of 36 days).

However, majority of hydrophobic cytotoxic agents cannot be introduced in the hydrogel matrix due to the hydrophilic character of the system. This constitutes the major limit to their use in antitumor therapy. To overcome this problem, hydrogels can be combined with nanosystems. During the last decades, nanovectors have been thoroughly studied as a therapeutic strategy against GBM (46, 47). Their size, composition and surface characteristics can be adjusted to deliver hydrophilic and/or lipophilic molecules to the brain cancer cells. Furthermore, they can protect the drugs against degradation, thus increasing their half-life while reducing toxic effects (47). But even improved by nanosystems, the BBB crossing is still too low after a systemic administration. The combination between nanoparticles and hydrogels is therefore an interesting strategy for the treatment of GBM. Indeed, these hybrid systems allow a simultaneous administration of multiple hydrophilic and/or lipophilic therapeutic active molecules in the tumor with a sustained release (41, 42, 48). Regarding GBM, these nanoparticle-loaded hydrogels can be injected in the tumor or implanted directly after the surgical resection. The latter is not well described in literature but constitutes a promising administration route.

### Liposome-Loaded Hydrogels and Glioblastoma

Only one pre-clinical study concerns the use of liposome-loaded hydrogels in the treatment of GBM. Indeed, Arai et al. have combined a thermoreversible gelling polymer with liposomes encapsulating doxorubicin (49). A sustained release of doxorubicin was observed (9, 24, 64 and 94% after 8, 14, 30 and 54 days, respectively) compared to non-encapsulated doxorubicin entrapped in the hydrogel (78, 94 and 100% after 2, 4 and 12 days, respectively). Intratumorally injected in a subcutaneous human GBM murine model (U-87 MG cell line), this hydrogel combined with liposomes encapsulating doxorubicin inhibited tumor growth up to 38 days after treatment, compared to 14 days with the hydrogels containing the non-encapsulated drug, showing promising avenue to have a long-term antitumor activity.

### Inorganic Nanoparticle-Loaded Hydrogels and Glioblastoma

Kim et al. studied the association between multifunctional nanoparticles combining antitumor effect and a real-time biodistribution tracking: CoFe_2_O_4_/SN-38 system, and a poly (organophosphazene) hydrogel (50). Hydrophobic interactions between the surface of the nanoparticles and the poly (organophosphazene) ethoxy L-isoleucine allowed the reticulation and the hydrogel formation at 37°C. *In vitro*, the release of CoFe_2_O_4_/SN-38 was observed over 59 days. On a subcutaneous human GBM model (U-87 MG cell line), no tumor growth was observed when intratumorally treated with the CoFe_2_O_4_/SN-38-loaded hydrogel (0.8 and 1.2 mg/mL) compared to the non-treated group and the groups treated with SN-38 alone, CoFe_2_O_4_ nanoparticle-loaded hydrogel and CoFe_2_O_4_/SN-38-loaded hydrogel (0.4 mg/mL). Using an orthotopic human GBM model (U-87 MG cell line), the treated zones were visible by MRI and the CoFe_2_O_4_/SN-38-loaded hydrogels (3.6 mg/mL) demonstrated a higher therapeutic effect compared to SN-38 in solution.

Meenach et al. (51) have developed iron oxide nanoparticles dispersed in a paclitaxel impregnated hydrogel. When the hydrogel is exposed to an external alternative magnetic field, it emits heat (41–45°C) very quickly and causes hyperthermia: another strategy used to treat the tumor cells with inorganic nanoparticles. This heat did not expand the hydrogel, suggesting that it will not increase the intracranial pressure. Paclitaxel *in vitro* release studies have shown a total release in 22 days. When GBM cells (M059K cell line) were exposed to heat (43°C) and paclitaxel (10 and 50 μM), the cell viability decreased. However, no synergistic effect between both elements was observed.

### Polymeric Nanoparticle-Loaded Hydrogels and Glioblastoma

Zhao et al. have studied poly (lactic-co-glycolic acid) nanoparticles encapsulating paclitaxel combined with a hydrogel reticulated by photopolymerization (52). Adding the polymer nanoparticles in the initial solution did not prevent the photopolymerization. Paclitaxel release from the hydrogel in phosphate buffered saline (PBS) is fast up to 8 h (11 ± 2%) and lasts for at least 7 days (29 ± 4%). Total paclitaxel release is supposed to happen after 4 weeks. *In vivo* short-term (2 months) and long-term (4 months) studies showed that non-loaded hydrogel did not induce apoptosis or increase microglia activation. The activity of paclitaxel, loaded in the polymeric nanoparticles themselves dispersed in the hydrogel, has been evaluated on a human GBM (U-87 MG cell line) murine resection model. Fifty percent of mice treated with this hydrogel are still alive 150 days after tumor cell inoculation whereas the median survival for paclitaxel-loaded polymeric nanoparticles alone (without hydrogel) or hydrogel alone (without paclitaxel-loaded polymeric nanoparticle) treated mice was 72 or 63.5 days, respectively. In another study, Zhao et al. added TMZ in the matrix of the paclitaxel-loaded hydrogel (53). A synergic effect of both drugs was observed *in vitro*. Using the same tumor resection model, the median survival for mice treated with TMZ-loaded hydrogel (without paclitaxel-loaded polymeric nanoparticle) or paclitaxel-loaded hydrogel (without TMZ) was 75 or 90 days, respectively, whereas the median survival of mice treated with both TMZ and paclitaxel-loaded hydrogel was still not defined after 110 days of experiment.

### Lipid Nanocapsule-Loaded Hydrogels and Glioblastoma

A hydrogel without polymer, only based on lipid nanocapsules, has been recently developed and used as a local treatment against GBM (54–57). The hydrogel formation is only due to the association of lipid nanocapsules in suspension using an amphiphilic crosslinking agent: 4-(N)-lauroyl gemcitabine (GemC12), located at the oil/water interface of lipid nanocapsules, forming hydrogen bonds between the gemcitabine moieties and so inter-nanoparticular interactions (54). The advantage of this system is that hydrogel degradation corresponds to the release of GemC12-loaded lipid nanocapsules because no other component (natural or synthetic polymer, gelling agents, etc.) is present in the hydrogel, compared to conventional nanoparticle-loaded hydrogels (48). In artificial cerebrospinal fluid, 56 ± 9% of GemC12-loaded lipid nanocapsules are released in the first 48 h. The lipid nanocapsule release is then slower and sustained for at least a month (77 ± 8% after 30 days) (55). Another advantage of this system is that once all lipid nanocapsules are released corresponding to the complete dissolution of the hydrogel, no implant residue remains at the implantation site. *In vivo*, this hydrogel was well tolerated in the healthy mouse brain at short and long-term (2 and 6 months, respectively) (55, 56). In addition, when the hydrogel was injected in the resection cavity right after the GBM (U-87 MG cell line) resection, the median survival of mice treated increased significantly compared to the non-treated mice (62 and 35.5 days, respectively) and the apparition of recurrences was delayed (56). This antitumor activity was also observed on a rat GBM orthotopic resection model (57).

Regarding the preclinical studies described above, there is no doubt about the benefits that hydrogels and more particularly nanoparticle-loaded hydrogels can provide for the local post-resection therapy strategies against GBM. These systems can be tailored using natural matrix for the hydrogels, controlling their viscoelastic properties to be as close as possible to the brain characteristics, and the large expertise of the scientists makes nanoparticles more and more stable, better and better tolerated and can be developed on a large scale. A wide range of therapeutic molecules can thus be considered, and the combination of intrinsic properties of nanoparticles and drugs opens the way to new strategies. Nevertheless, the second limitation of Gliadel^®^ wafers is the lack of specificity of the treatment, and simple nanoparticle-loaded hydrogel also have this disadvantage. A common strategy used in nanovectorization is the combination of a targeting ligand on the surface of nanoparticles. The combination of these specific nanoparticles and the local administration inside a hydrogel seem quite easy to achieve but not yet tested. It is important here to review how or by which ligand to target GBM.

## Glioblastoma Targeting

The aggressiveness of GBM is characterized by its strong ability to proliferate and invade healthy tissues, properties due to the high heterogeneity of the GBM. These characteristics, as well as the intra- and inter-patient variabilities in GBM, make tumor cells very difficult to target and eliminate completely without affecting healthy surrounding cells (58). It is thus important to propose therapeutic strategies that could improve the targeting of different elements such as molecular biomarkers involved in the development of GBM. This targeting would allow a better drug delivery in the tumor and thus a decrease of side effects due to the molecule cytotoxicity.

The tumor microenvironment is composed of several cell types including cancer and cancer stem cells, endothelial cells, fibroblasts, perivascular and inflammatory cells, surrounded by extracellular matrix (ECM). These elements are involved in the control of cell growth, homeostasis but also in the regulation of the tumorigenic process (angiogenesis, lymphangiogenesis and inflammation) (59, 60). Immune cells can promote the development of cancer and associated disease processes. ECM is also likely to influence the growth of malignant cells by releasing proteins, growth factors, cytokines and enzymes that allow the motility and adhesion of cancer cells (61, 62). To alleviate the passive targeting limits, ligands can be added to the surface of nanoparticles in order to increase their selective absorption and accumulation in the targeted tissues. The benefit of this approach is that some receptors are often overexpressed on the surface of the cancer cell and other cells involved in the tumor microenvironment, and/or not expressed on healthy cells. The strategies described below have been used with intratumoral or systemic administrations but could be easily associated with drug delivery systems with drug sustained release profiles. Tables 1–4 summarize the different strategies to target the GBM cells, the GBM cancer stem cells, the tumor-associated myeloid cells and the extracellular matrix, respectively.

**Table 1:** Selection of ligands for GBM cell targeting, with the corresponding receptors.

**Ligand^a^**	**Receptors^a^**	**Nanoparticles**	**Cellular model^b^**	**References**
CTX	CIC-3	Polymer	*In vitro* (U87-MG and GI-1)	(63)
CTX	CIC-3	Biopolymer	*In vitro* (U118-MG, SF767 and GBM6)	(64)
CTX	CIC-3	Iron oxide	*In vitro* (T98G) *In vivo* (Ectopic T98G / IV)	(65)
CTX	CIC-3	Iron oxide	*In vitro* (SF767) *In vivo* (Orthotopic GBM6-luc / IT)	(66)
CTX	CIC-3 / MMP-2	Iron oxide	*In vitro* (U87-MG and T98G) *In vivo* (Orthotopic T98G / IT)	(67)
CTX	CIC-3 / MMP-2	Silver	*In vitro* (U87-MG, A172 and T98G) *In vivo* (Orthotopic U87-MG and U87-MG luc2 / IP)	(68)
CTX	MMP-2	Silver	*In vitro* (U87-MG) *In vivo* (Ectopic U87-MG / IV)	(69)
HA	CD44	Liposome	*In vitro* (A172, U251, U87-MG)	(70)
HA	CD44	Lipid	*In vitro* (T98G, U251 and U87-MG) *In vivo* (Orthotopic U87-MG / IT)	(71)
Anti-EGFRvIII antibody	EGFRvIII	Polymer	*In vitro* (DKMG/EGFRvIII and DKMG^low^)	(72)
Anti-EGFRvIII antibody	EGFRvIII	Iron oxide	*In vitro* (U87-MG and U87wtEGFR) *In vivo* (Orthotopic U87wtEGFR / IT)	(73)
Cetuximab	EGFRvIII	Iron oxide	*Ex vivo* (primary tumor) *In vivo* (Orthotopic primary tumor, U87wtEGFR and LN229wtEGFR / IT)	(74)
Anti-EGFR and anti-EGFRvIII antibodies	EGFR	Iron oxide	*In vitro* (U87-MG-EGFRvIII) *In vivo* (Orthotopic U87-MG-EGFRvIII / IT)	(75)
Anti-EGFR antibody	EGFR	Solid lipid	*In vitro* (U87-MG)	(76)
Cetuximab	EGFR	Polymer	*In vitro* (U87-MG and LN229) *In vivo* (Orthotopic U87-MG / IV)	(77)
EGF / Tf	EGFR	Gold	*In vitro* (U87-MG and LN229) *In vivo* (Orthotopic U87-MG / IV)	(78)
EGFR-binding peptide	EGFR	Biopolymer	*In vitro* (U87-MG) *In vivo* (Orthotopic U87-MG / IV)	(79)
RGD peptide	Integrin αvβ3 receptor	Biopolymer	*In vitro* (U87-MG) *In vivo* (Orthotopic U87-MG / IV)	(79)
RGD peptide	Integrin αvβ3 receptor	Silica	*In vitro* (U87-MG, U231 and C6)	(80)
*Gracilaria lemaneiformis* polysaccharide	Integrin αvβ3 receptor	Selenium	*In vitro* (U87-MG and C6)	(81)
IL-13 (Chimeric Antigen Receptor T cells)	IL-13 receptor α2	Polymer	*In vitro* (U87-MG luc) *In vivo* (Orthotopic U87-MG luc / IV)	(82)
Pep-1 peptide	IL-13 receptor α2	Polymer	*In vitro* (U87-MG) *In vivo* (Orthotopic U87-MG / IT)	(83)
ITEM4 antibody	Fn14	Polymer	*In vitro* (U87-MG luc) *In vivo* (Orthotopic U87-MG luc / IT)	(84)
ITEM4 antibody	Fn14	Polymer	*In vitro* (KR158 and KR158 luc) *In vivo* (Orthotopic KR158 luc / IT)	(85)

*The nature of nanoparticles as well as the type of preclinical studies and the cellular models are also reported*.

a *CTX, chlorotoxin; HA, hyaluronic acid; EGFR(vIII), epidermal growth factor receptor (variant III); EGF, epidermal growth factor; Tf, transferrin; IL-13, interleukin 13; CIC-3, chloride channel; MMP-2, matrix metalloproteinase 2; CD44, transmembrane glycoprotein; Fn14, fibroblast growth factor-inducible 14*.

b *IV, intravenous; IT, intratumoral; IP, intraperitoneal*.

**Table 2:** Selection of ligands for GBM cancer stem cell targeting, with the corresponding receptors.

**Ligand^a^**	**Receptors^a^**	**Nanoparticles**	**Cellular model^b^**	**References**
Anti-CD133 antibody	CD133	Polymer (dendrimer)	*In vitro* (SU2, GSC-derived U87-MG) *In vivo* (Orthotopic SU2 / IT)	(86)
Anti-CD133 antibody	CD133	Silica	*In vitro* (GSC)	(87)
Anti-Nestin antibody	Nestin	Iron oxide	*In vitro* (GSC-derived U251-MG) *In vivo* (Orthotopic GSC-derived U251-MG / IV)	(88)
Nestin-binding peptide	Nestin	Gold	*In vitro* (X01)	(89)
Nestin-binding peptide	Nestin	Gold	*In vitro* (X01)	(90)

*The nature of nanoparticles as well as the type of preclinical studies and the cellular models are also reported*.

a *CD133, transmembrane glycoprotein*.

b *IV, intravenous; IT, intratumoral*.

**Table 3:** Selection of ligands for tumor-associated myeloid cell targeting, with the corresponding receptors.

**Ligand^a^**	**Receptors^a^**	**Nanoparticles**	**Cellular model^b^**	**References**
Anti-PD-L1 antibody	PD-L1	Liposome	*In vitro* (GL261 and TAMs generated form CT2A and GL261 culture medium) *Ex vivo* (TAMs isolated from primary tumor or orthotopic GL261) *In vivo* (Orthotopic GL261 + TAMs / IT)	(91)
Cross-talk between macrophages (carrier) and GBM cells (target)		Diamond	*In vitro* (histiocytic lymphoma U937 cells) *In vivo* (Orthotopic U87-MG and GL261 + allograft mouse bone marrow-derived macrophages / IV)	(92)
(NOX-E36) CCL2 inhibitor	CCL2	NOX-E36 (PEGylated active agent)	*In vitro* (U87-MG/CCL2^+^ and LN18/CCL2^+^) *In vivo* (Orthotopic U87-MG/ CCL2^+^ / IP)	(93)

*The nature of nanoparticles as well as the type of preclinical studies and the cellular models are also reported*.

a *PD-L1, programmed death protein 1; CCL2: chemokine ligand*.

b *TAMs, tumor-associated macrophages; IT, intratumoral; IV, intravenous; IP, intraperitoneal*.

**Table 4:** Selection of ligands for extracellular matrix targeting, with the corresponding receptors.

**Ligand^a^**	**Receptors^a^**	**Nanoparticles**	**Cellular model^b^**	**References**
CTX	MMP-2	Polymer	*In vitro* (U87-MG and GI-1)	(63)
CTX	MMP-2	Polymer	*In vitro* (U87-MG, A172 and T98G) *In vivo* (Orthotopic U87-MG / IP)	(68)
CTX	MMP-2	Silver	*In vitro* (U87-MG) *In vivo* (Orthotopic U87-MG / IV)	(69)
MMP-cleavable peptide	MMP-2 / MMP-9	Lipid	*In vitro* (U87-MG and bEnd.3)	(94)
Sulfatid	Tenascin C	Liposome	*In vitro* (U118-MG) *In vivo* (Ectopic U118-MG / IV)	(95)
FHK peptide	Tenascin C	Polymer	*In vitro* (U87-MG) *In vivo* (Orthotopic U87-MG / IV)	(96)
tLyp-1 peptide	Neuropilin	Polymer	*In vitro* (C6) *In vivo* (Orthotopic C6 / IV)	(97)
F3 peptide	Neuropilin	Polymer	*In vitro* (C6) *In vivo* (Orthotopic C6 / IV)	(98)
Lactoferrin	Neuropilin	Polymer	*In vitro* (C6) *In vivo* (Orthotopic C6 / IV)	(99)
PL3 peptide	Tenascin C / Neuropilin	Iron oxide / Silver	*In vitro* (U87-MG and murine wt GBM) *In vivo* (Orthotopic murine wt GBM / IV)	(100)
LinTT1 peptide	Neuropilin / p32 protein	Iron oxide / Silver / Biopolymer	*In vitro* (U87-MG, wt GBM and VEGF KO GBM) *In vivo* (Ectopic U87-MG and orthotopic wt GBM and VEGF KO GBM / IV)	(101)
CREKA peptide	Fibrin-fibronectin complex	Polymer	*In vitro* (U87-MG) *In vivo* (Orthotopic U87-MG / IV)	(83)
CREKA peptide	Fibrin-fibronectin complex	Iron oxide	(Murine myocardial ischemia / reperfusion model)	(102)

*The nature of nanoparticles as well as the type of preclinical studies and the cellular models are also reported*.

a *CTX, chlorotoxin; MMP(-2/-9), matrix metalloproteinase (2/9)*.

b *IP, intraperitoneal; IV, intravenous; wt, wildtype; VEGF KO, vascular endothelial growth factor knockout*.

### Glioblastoma Cell Targeting

Several molecules are found overexpressed in GBM cells. Among these molecules are the chloride channels, particularly CIC-3. One of the most used molecules to target CIC-3 on the surface of GBM cells is chlorotoxin (CTX), a highly specific scorpion venom peptide. Surface functionalization of the nanoparticles with CTX has been used to increase the efficacy of therapeutic assets (63–65, 68, 69). For example, Yoo et al. (67) developed iron oxide nanoparticles as a vector of O6-methylguanine-DNA methyltransferase specific interfering RNA (siMGMT), one of the molecules responsible for tumor cell resistance to TMZ. These nanoparticles were functionalized on their surface with CTX (CTX-NP-siMGMT), which increased *in vitro* nanoparticle uptake by GBM cells (U-87 MG and T98G cell lines), compared to non-functionalized nanoparticles (NP-siMGMT). However, this absorption has not been tested on healthy cells. The addition of siMGMT promoted the suppression of expression and activity of O6-methylguanine-DNA methyltransferase in GBM cells (T98G cell line), leading to higher cell sensitization to TMZ. These effects were also observed when mice with orthotopic human GBM (T98G cell line) were intratumorally treated with CTX-NP-siMGMT. The tumor size was also reduced when mice were treated with CTX-NP, probably due to the anti-proliferative activity of CTX. Indeed, when binding to the chloride channels, CTX disturbs the chloride gradients essential for migration and the invasive character of GBM cells. In the same vein, Stephen et al. developed iron oxide nanoparticles functionalized with CTX to deliver 6-O-benzylguanine, a O6-methylguanine-DNA methyltransferase inhibitor, to GBM cells (after intratumoral administration in GBM orthotopic model) (66).

CD44 is a receptor located on the surface of cell membranes and is involved in cell-to-cell interactions (103). The expression of this glycoprotein is greater in several cancers, including GBM, compared to healthy tissues (104–107). In addition, studies have shown that this receptor is involved in the invasion of tumor cells (108, 109). Hyaluronic acid (HA), a major component of extracellular matrix, is a natural ligand of CD44 used to target cancer cells overexpressing the receptor thus improving its specificity and efficacy (110–113). Hayward et al. (70) have shown that surface-conjugated liposomes with HA specifically target human GBM cells (A172 and U-87 MG cell lines) compared to primary astrocytes and rat microglia cells. In addition, this system has improved the therapeutic efficacy of doxorubicin encapsulated in liposomes, while decreasing its absorption by astrocytes and microglia cells. Cohen et al. (71) also showed that lipid nanoparticles functionalized with HA could bind to GBM cells (U-87 MG cell line) but also to neurospheres of GBM cells from patients. These lipid nanoparticles charged with an interfering RNA directed against Polo-Like Kinase 1 (siPLK1), a kinase involved in cell cycle regulation, significantly reduced the expression of PLK1 mRNA in U-87 MG cells compared to lipid nanoparticles charged with siPLK1 but without HA on their surface. In addition, this expression decrease was correlated with higher cell toxicity. Finally, *in vivo* studies using an orthotopic human GBM model (U-87 MG cell line) showed that intratumoral treatment with lipid nanoparticles combined with HA and siPLK1 increased the mouse median survival compared to saline and lipid nanoparticles combined with HA and siLuciferase (not determined after 95 days compared to 33 and 34.5 days, respectively).

Epidermal growth factor receptor (EGFR) is part of the tyrosine kinase receptor family. This transmembrane glycoprotein is found overexpressed in about 40% of primary GBM (114, 115), and is involved in cell proliferation and survival (116, 117). The most common mutation of the EGFR gene is variant III (EGFRvIII), constitutively activated, and accounts for up to 60% of EGFR amplifications in primary GBM (118). This characteristic makes it a preferential target (119), and many laboratories developed nanomedicines using anti-EGFR and anti-EGFRvIII antibodies in combination with many types of nanoparticles to preferentially target these receptors. For example, Jamali et al. (72) conjugated a monoclonal antibody anti-EGFRvIII to PLGA nanoparticles encapsulating curcumin used as a photosensitizer for the development of photodynamic therapy on GBM cells. Others also used cetuximab, an antibody recognizing EGFR and EGFRvIII and inhibiting their action, or other types of ligands, such as an EGF peptide, in combination with iron oxide (73–75), gold (78), polymeric (77), solid lipid (76) and human ferritin nanoparticles (79).

Other molecules are also found deregulated in GBM cells such as the integrin αvβ3 receptor, the α2 receptor of IL-13 or the fibroblast growth factor-inducible 14 (Fn14). The integrin αvβ3 receptor is a cell adhesion molecule that plays a role in cell propagation, migration, survival, proliferation, and differentiation (120). It is found overexpressed in glioma cells and newly formed vessels, thus also an interesting target for the development of nanomedicines (121). The cyclic peptide RGD was used in combination with several types of nanoparticles (human ferritin ones or mesoporous silica) to target this receptor in the treatment of GBM (79, 80). In another study, Jiang et al. (81) functionalized selenium nanoparticles with polysaccharides from the *Gracilaria lemaneiformis* alga which have a strong affinity for the integrin αvβ3 receptor. Interleukin 13 (IL-13) is a cytokine involved in the regulation of immune responses and microenvironment. In most cells, IL-13 binds with low affinity to the α1 receptor (IL-13Rα1) which then pairs to the α receptor of interleukin 4 to form a heterodimer. This complex then enables the activation of STAT6 signaling pathways. In some healthy cells and in cancer cells, IL-13 can bind with strong affinity to the α2 receptor (IL-13Rα2). IL-13Rα2 is a decoy receptor that sequesters IL-13, allowing tumor cells to escape apoptosis (122). This receptor is found overexpressed in 75% of GBM (123). Kim et al. (82) grafted doxorubicin-loaded nanoparticles to the T lymphocyte surface with a mutant version of IL-13. Wang et al. (83) functionalized the surface of PLGA nanoparticles with the Pep-1 peptide, specifically recognizing IL-13Rα2. Fn14 is a member of the tumor necrosis factor receptor family. Its expression level in the healthy brain is low whereas it is found more important in 70 to 85% of GBM (124). Moreover, the overexpression of Fn14 was found in both primary tumor and tumor cells infiltrated in healthy tissue (124, 125), whether newly diagnosed or recurrent (126, 127). These data make Fn14 an optimal surface molecule for the development of targeting therapy using nanoparticles associated with anti-Fn14 antibodies for the treatment of GBM (84, 85).

### Glioblastoma Cancer Stem Cell Targeting

The GBM cancer stem cells (CSC), resistant to radio and chemotherapy are tumor-initiating cells, also partly responsible for the recurrences observed in patients after the implementation of standard treatments (128). Therefore, the targeting and eradication of CSC could lead to a better management of GBM. GBM CSC can express biomarkers associated with neural stem cells (129–132), and these biomarkers have been used as targets in several studies to improve drug delivery (87–90).

Among the markers that can be associated with CSC, CD133 is one of the most studied in brain tumors (133). This membrane glycoprotein plays a role in cell differentiation and the epithelial-mesenchymal transition. In the 2000's, studies have shown that cells with the marker CD133, isolated from human brain tumors, can reproduce the original tumor in immunocompromised mice (134, 135). Furthermore, recurrences of GBM often have a higher percentage of CD133-positive cells compared to tumor cells before treatment. A large proportion of CD133-positive cells is correlated with lower patient survival (136). These cells with a high capacity to form tumors have therefore become targets for the treatment of GBM (86, 87). However, not all GBM CSC express CD133. Subsequent studies have shown that tumors can successfully develop from GBM CSC without the CD133 marker in xenograft models (137, 138). Therefore, the identification of GBM CSC cannot be based solely on the expression of CD133. CD133-positive cells often co-express nestin, a protein from one of the cytoskeletal components (intermediate filaments). The expression of nestin is found in neural and progenitor stem cells but also in several types of cancer, including GBM (134, 135, 139–141). Increased expression of nestin is associated with high-grade gliomas and a low patient survival rate (132, 141).

To compensate for this low survival, several teams were interested in the treatment of GBM CSC. For example, Prabhu et al. (88) developed TMZ-charged iron oxide nanoparticles functionalized with an anti-nestin antibody to fight GBM CSC while sparing healthy tissue. In addition, Gonçalves et al. (89, 90) developed gold nanorods (AuNR) combined with a peptide specifically recognizing nestin (NesPEG-AuNR). These AuNR generate heat when irradiated by a laser emitting in the near infrared and cause localized cellular damage. *In vitro* internalization studies using cells expressing or not nestin, cultured in single layer (2D culture) or spheroids (3D culture) showed that NesPEG-AuNR were mainly internalized by nestin-positive cells and not by nestin-negative cells. Internalization of AuNR involves energy-dependent mechanisms, including endocytosis mediated by caveolin. Photothermal treatments of NesPEG-AuNR resulted in selective elimination of nestin-positive cells by cell apoptosis, while nestin-negative cells remained viable. The results also indicated that in the presence of AuNR, nestin-positive cells as spheroids are more resistant to photothermal treatments than when they are cultured in a monolayer, indicating that the 3D model is closer to *in vivo* models than the 2D model.

### Tumor-Associated Myeloid Cell Targeting

Tumor-associated myeloid cells (TAMCs) are a heterogeneous population of myeloid cells from hematopoietic precursors. They include tumor-associated macrophages (TAMs) and myeloid-derived suppressor cells (MDSCs). TAMCs are massively recruited at the GBM level, reaching 30 to 50% of the tumor mass (142–144). These cells are the main cause of immunosuppression in GBM (60, 145, 146). They can strongly inhibit innate and adaptive immunity (147–149). They suppress effective immune cell function by several pathways, especially by depriving lymphocytes from their essential nutriments, generating oxidative stress and triggering the recruitment of regulatory T cells (147). As a result, TAMCs have recently been recognized as an attractive therapeutic target to decrease immunosuppression in GBM in the hope of maximizing the effectiveness of anti-tumor therapies.

TAMCs have recently been shown to express the programmed death-ligand 1 (PD-L1) more strongly than other immune cells or tumor cells (150, 151). This advantage was used by Zhang et al. (91) to develop surface functionalized lipid nanoparticles with anti-PD-L1 antibody. *Ex vivo*, the nanoparticles combined with the antibody were able to target TAMCs, to be internalized and to accumulate at the level of lysosomes. These functionalized nanoparticles encapsulating dinaciclib, a cyclin-5-dependent kinase inhibitor, reduced the viability of TAMCs in a dose-dependent manner. In addition, they reduced the recycling of PD-L1 on the surface of TAMCs by directing the ligand to the lysosome. In an orthotopic *in vivo* model of murine GBM (GL261 cell line), 24 h after injection at different locations in the brain, the nanoparticles functionalized with the anti-PD-L1 antibody encapsulating dinaciclib were retained in the tumor and more particularly, co-located with TAMCs. In addition, the median survival was significantly higher in mice treated with radiotherapy combined with these nanoparticles compared to mice treated with saline solution, radiotherapy alone and nanoparticles alone. These results were also observed when mice were intranasally treated. Finally, these nanoparticles were able to target TAMCs from GBM patients, validating the relevance of this approach on a human model.

Monocytes recruited in cancer tissues differentiate into macrophages that can be activated into two different phenotypes (type 1 or 2) in response to signals from their microenvironment. Type 1 macrophages (M1) coordinate the development of an adverse inflammatory microenvironment for cancer cells and play a central role in initiating and maintaining antitumoral immunity. On the contrary, type 2 macrophages (M2) suppress anti-tumor immunity and coordinate the remodeling of the microenvironment, making it favorable to survival, growth and tumor progression. Many studies suggest that TAMs are primarily M2-type in GBM (152, 153). Strategies to reduce the number of TAMs in the tumor or modulate their phenotype have shown strong potential for the treatment of GBM (154, 155).

Li et al. used the intertwined relationships between TAMs and GBM cells to modify the M2 phenotype of TAMs (92). TAMs with an M2 phenotype, loaded with nanodiamonds combined with doxorubicin (Nano-DOX) were able to deliver the Nano-DOX to GBM cells *in vitro* and *in vivo*, causing damage to these cells. The altered GBM cells emitted molecular patterns associated with this damage, modifying the M2 phenotype of the TAMs to a M1 phenotype and thus reducing the tumor size of a human orthotopic GBM model (U-87 MG cell line). Chemokine ligand 2 (CCL2) is involved in differentiation and survival of TAMs. Cho et al. used a CCL2 inhibitor (NOX-E36) to suppress their recruitment and study the effect of combined therapy with bevacizumab, an antibody directed against the vascular endothelium growth factor (93). Inhibition of CCL2 blocked macrophage recruitment and angiogenesis, resulting in decreased tumor and blood volumes in an orthotopic human GBM model (U-87 MG cell line) expressing CCL2. In addition, the median survival of mice treated with NOX-E36 combined with bevacizumab is greater than that of mice treated with bevacizumab alone (32 and 22 days, respectively). This study shows that CCL2 inhibition can play an important role in increasing the effectiveness of anti-angiogenic treatment in GBM by inhibiting the recruitment of TAMs.

### The Extracellular Matrix Targeting

The extracellular matrix (ECM) consists of a complex network of fibronectins, collagens, chondroitins, laminins, glycoproteins, heparin sulfate, tenascins and proteoglycans (156). These molecules play a role in migration, differentiation and inflammation but they also participate in the process of invasion and metastasis of malignant cells in the host tissue (157). Some of these molecules are found at high levels in ECM of tumor tissue. This feature has been used to target and improve drug delivery in the GBM. In this next section, only the most studied proteins for the treatment of GBM in combination with NP have been described.

ECM is characterized by the presence of matrix metalloproteinases (MMP), enzymes part of the gelatinase family, which play a key role in tumor progression and metastasis. In GBM, MMP-2 and MMP-9 are overexpressed. They degrade ECM and contribute to the angiogenic and invasive potential of glioma cells. These characteristics make the MMP attractive targets for the development of GBM therapy (63, 158). Agarwal et al. (63) developed morusin (MOR)-loaded PLGA nanoparticles, a naturally occurring chemotherapy active ingredient, and surface-conjugated with CTX (PLGA-MOR-CTX). The anti-cancer potential of PLGA-MOR-CTX nanoparticles was evaluated *in vitro* in human GBM cells (U-87 MG and GI-1 cell lines). PLGA-MOR-CTX nanoparticle treatment resulted in nanoparticle accumulation in GBM cells, mediated by MMP-2 targeting. In addition, important cytotoxicity parameters such as reactive oxygen species generation, increased caspase activity, cytoskeletal destabilization, and inhibition of MMP-2 activity were observed in GBM cells following treatment with PLGA–MOR–CTX nanoparticles. These results, combined with the non-toxicity of healthy human neuronal cells (HCN-1A cell line), highlight the specific therapeutic potential of this strategy for the treatment of GBM. Other teams have also targeted MMP-2 to deliver therapeutic nanoparticles in GBM (68). For example, Locatelli et al. (69) formulated multifunctional nanocomposites consisting of polymeric nanoparticle containing two cytotoxic agents: alisertib, an inhibitor of Aurora A kinase, and silver nanoparticles conjugated with CTX. In addition, Bruun et al. (94) used lipid nanoparticles loaded with a siRNA and functionalized with angiopep-2 and a lipopeptide capable of targeting MMP to target ECM.

Tenascin C (TNC) is a glycoprotein of ECM overexpressed during normal tissue repair and in many malignant tumors. It plays an important role in tumor progression including angiogenesis, proliferation and cell migration, making it an attractive target for GBM therapy (159–161). Doxorubicin-loaded liposomes, modified with sulfatid known to bind to TNC, have been used to improve efficacy and reduce the side effects of free doxorubicin (95). Biodistribution, therapeutic efficacy and systemic toxicity of the liposomes were evaluated in an *in vivo* human GBM xenograft model (U-118 MG cell line). The median survival was greater in GBM-bearing mice treated with liposomes compared to free doxorubicin and saline (93, 61 and 45 days, respectively). Kang et al. adopted a different strategy by targeting both TNC and neuropilin-1 (NRP-1), a transmembrane protein overexpressed in newly formed tumor cells and blood vessels (96). The FHK peptide targeting TNC was coupled with the tLyp-1 peptide known to increase penetration of nanosystems into tumor cells via NRP-1 (97–99). PLA nanoparticles loaded with paclitaxel were functionalized with this FHK/tLyp-1 peptide (FHK/tLyp-1-NP-PTX). *In vitro*, the functionalization of the nanoparticles allowed their internalization in U-87 MG and HUVEC cells (two-dimensional culture), but also their deep penetration into GBM spheroids. In addition, *in vivo*, real-time imagery showed an accumulation of functionalized nanoparticles in GBM using an orthotopic mouse model (U-87 MG cell line). It has been shown that this accumulation is mediated by TNC and that transport through cancer cells is governed by NRP-1. Finally, the median survival of the mice was greater when treated with FHK/tLyp-1-NP-PTX compared to FHK-NP-PTX, tLyp-1-NP-PTX, NP-PTX and PTX (59 compared to 37.5, 33.5, 25.5 and 16 days, respectively). These results suggest that the combination of the two peptides provides an interesting tool both to target GBM and to treat it using a synergistic mechanism. The targeting of TNC or one of its isoforms in combination or not with other molecules to improve drug delivery was also explored (100, 101).

Fibronectin (FN) is a glycoprotein that plays a role in cell adhesion, migration, growth and differentiation. This protein can bind to several molecules including fibrin. Fibrin-FN complexes play a role in coagulation (162). The significant presence of fibrin-FN complexes is found in many invasive tumors, including GBM (163). These complexes play an important role in survival, the proliferation and invasion of cancer cells, making it another attractive target for the treatment of GBM (164). Wang et al. (83) functionalized PLGA nanoparticle surface with the Pep-1 and CREKA peptides (Pep-1/CREKA-NP). Pep-1 peptide can pass through the BBB and penetrate GBM cells by targeting the IL-13Rα2, overexpressed on the plasma membrane of GBM cells (123). CREKA peptide works as an anchor by binding to fibrin-FN complexes in the ECM of tumor cells (102). The functionalization of the nanoparticles with both CREKA and Pep-1 increased the nanoparticle retention in the GBM tissue (U-87 MG cell line) and the distribution of the therapeutic agent in cancer cells. In fact, Pep-1/CREKA-NP penetrated deeper into GBM compared to nanoparticles combined with any of the peptides (Pep-1-NP or CREKA-NP). In addition, the median survival of mice with orthotopic human GBM (U-87 MG cell line) treated with Pep-1/CREKA-NP was higher than those treated with Pep-1-NP, CREKA-NP, NP-PTX, PTX and a saline solution (61 compared to 53, 55, 47, 43 and 36 days, respectively).

## Conclusion

GBM represents 240,000 new cases per year worldwide and its standard of care has not evolved for too many years. As previously reported, GBM is one of the most expensive cancers to treat, which is all the more paradoxical since these treatments are not curative. After initial surgical resection, poor prognosis (median survival of 14 months, 5 year survival <10%) is ascribed to frequent recurrences in proximity of the original tumor. Thus, there is an unmet medical need for better treatments. On societal and economic levels, improved survival and increased efficacy of costly treatments could positively impact public health and social security. Better control over the growth of GBM could also improve the quality of life of patients and it will promote social and professional reinsertion and reduce indirect costs associated with the disease. Nevertheless, surgery is still essential when possible, ensuring a prolongation of the patient life expectancy. Thus, it is interesting to take advantage of this surgical act to develop post-resection implants, and to bridge the treatment gap between surgical resection and initiation of conventional radio- and chemotherapy, and possibly lowering the risks of recurrences. For patients, a pro-active strategy might be better accepted than the current period of non-treatment, a “wait-and-see” approach consisting of waiting for the Stupp protocol within a post-surgical period which can vary according to the patient. Regarding the design of the implants, the nanoparticle-loaded hydrogels are a promising strategy because (i) their rigidity can be adjusted to the brain elasticity reducing the side effect, and (ii) the sustained release of the nanoparticles, loaded with anticancer agents, will provide a continuous treatment. In addition, the use of nanoparticles will offer the possibility of active tumor targeting through ligands at their surface. Due to GBM complexity, the possibility of multi-targeting, with ligands specific to different elements of the tumor microenvironment as a whole, could provide a synergy of treatments. However, it should be kept in mind that despite improvements in neurosurgical techniques, not all diagnosed GBMs are necessarily operable. So, the implant-based GBM therapy is not an option for these patients. Other strategies such as crossing or by-passing the BBB or the blood-cerebrospinal fluid barriers, must be concomitantly implemented. This combination of local, sustained and targeting drug delivery remains to be explored and could also be considered for other pathologies requiring such a therapeutic scheme.

## Author Contributions

CG and MS wrote the original manuscript, have contributed equally to this work and share first authorship. PS, JE, and GB reviewed the manuscript. GB was the one who initiated the funding request, administrated the project, supervised the work and edited the manuscript. All authors contributed to the article and approved the submitted version.

## Conflict of Interest

The authors declare that the research was conducted in the absence of any commercial or financial relationships that could be construed as a potential conflict of interest.

## Publisher's Note

All claims expressed in this article are solely those of the authors and do not necessarily represent those of their affiliated organizations, or those of the publisher, the editors and the reviewers. Any product that may be evaluated in this article, or claim that may be made by its manufacturer, is not guaranteed or endorsed by the publisher.
